# Magnitude of tuberculosis lymphadenitis, risk factors, and rifampicin resistance at Adama city, Ethiopia: a cross-sectional study

**DOI:** 10.1038/s41598-023-43206-7

**Published:** 2023-09-24

**Authors:** Hawi Kumbi, Dawit Yihdego Reda, Manyahlehal Solomon, Alemwosen Teklehaimanot, Moges Desta Ormago, Musa Mohammed Ali

**Affiliations:** 1 Department of Laboratory, Adama Hospital Medical College, Adama, Ethiopia; 2 School of Medical Laboratory Science, Hawassa University College of Medicine and Health Sciences, Hawassa, Ethiopia; 3 Department of Pathology, Adama Comprehensive Specialized Hospital Medical College, Adama, Ethiopia; 4 Department of Pathology, Hawassa University College of Medicine and Health Sciences, Hawassa, Ethiopia

**Keywords:** Microbiology, Medical research

## Abstract

*Mycobacterium tuberculosis* complex has an impact on public health and is responsible for over one million deaths per year. Substantial numbers of people infected with *M. tuberculosis* can develop tuberculosis lymphadenitis; however, there is a limited study in Adama, Ethiopia. The aim of this study was to determine the magnitude of Tuberculosis lymphadenitis, its predictors, and rifampicin-resistance gene-positive *M. tuberculosis*. A total of 291 patients with enlarged lymph nodes were recruited from May 2022 to August 30 at Adama Comprehensive Specialized Hospital Medical College (ACSHMC). GeneXpert, Ziehl–Neelsen staining, and cytology were used for the diagnosis of TB lymphadenitis from the Fine Needle Aspirate (FNA) specimen. Rifampicin-resistant gene was detected using GeneXpert. For data entry and analysis, Epi Data version 3.0 and SPSS version 25 were used respectively. A binary logistic regression model was used to identify predictors of TB lymphadenitis. A *p* < 0.05 with a 95% confidence interval (CI) was taken as a cut point to determine the significant association between dependent and independent variables. The prevalence of TB lymphadenitis using GeneXpert, Ziehl–Neelsen staining, and cytology were 138 (47.4%) (95% CI 41.70–53.10), 100 (34.4%) (95% CI 28.94–39.85), and 123 (42.3%) (95% CI 36.63–47.00) respectively. Nine (3.1%) participants were infected with rifampicin-resistant gene*-*positive *M. tuberculosis*. Out of the total *M. tuberculosis* detected by GeneXpert (n = 138), 9 (6.5%) were positive for rifampicin resistance-gene. Participants with a chronic cough had 2 times odds of developing TB lymphadenitis (AOR: 2.001, 95% CI 1.142–3.508). Close to half of patients with enlarged lymph nodes were positive for *M. tuberculosis* by the GeneXpert method in the study area. Chronic cough was significantly associated with TB lymphadenitis. Rifampicin-resistant gene-positive *M. tuberculosis* was relatively prevalent among patients with enlarged lymph node in the study area.

## Introduction

Tuberculosis is one of the leading causes of death, killing 2 million people each year^1^. TB lymphadenitis is the most common type of extrapulmonary tuberculosis that occurs outside of the lungs^2^. According to Stop TB Partnership, Ethiopia is among 30 countries suffering from high tuberculosis burden. In Ethiopia, about one-third of cases of tuberculosis are attributed to TB lymphadenitis^3^.

Lymphadenitis refers to lymph nodes that are abnormal in size, number, or consistency and can have etiologic agents ranging from infectious processes to malignant disease. The cause of lymphadenitis is difficult to diagnose based on history and physical examination alone. Fine needle aspiration cytology (FNAC) is a widely accepted, cost-effective, and safe method for the diagnosis of TB lymphadenitis and other lymph node abnormalities^4^.

TB lymphadenitis is a chronic specific granulomatous inflammation of the lymph node with caseous necrosis caused by infection with *Mycobacterium tuberculosis* or related bacteria. T cell lymphocytes and fibroblasts granulomatous tuberculosis eventually lead to the development of central caseous necrosis and a tendency to merge with the replacement of lymphoid tissue^5^. TB lymphadenitis (cervical) can be caused by the spread of *M. tuberculosis* from a lung infection^6^.

Currently, lymphadenitis is a common pathological problem in the world. Many studies have been conducted to determine the magnitude and etiology of lymphadenitis. The pattern of the disease varies across different ethnic backgrounds and countries. Information regarding the magnitude and etiology of lymphadenitis in a specific geographic area is essential for a better diagnosis, treatment, and control of the disease^7^.

According to a study conducted in Ethiopia, 196 *M. tuberculosis* isolated from enlarged lymph nodes belongs to different lineages^8^. In a review and meta-analysis conducted in Africa, a total of 6746 TB lymphadenitis cases were identified. The majority of the cases (70.6%) were from Ethiopia. Over 77% and 88% of identified TB lymphadenitis involved the cervical region and did not receive anti-TB drugs^9^.

In addition to TB lymphadenitis, there are various causes of pathologies of lymph nodes; the most common are malignant tumor, reactive hyperplasia, Hodgkin lymphoma, non-Hodgkin lymphoma, purulent abscess, and other chronic inflammation. Lymph nodes have a characteristic presentation depending on the etiology and can present as acute painful swelling due to infection or chronic painless swelling^7,10,11^.

Even though Ethiopia is among high TB burden countries, there is limited data of TB lymphadenitis from Adama, East Shoa zone, Oromia Regional State. In this study, we aimed to determine the magnitude of TB lymphadenitis among patients with enlarged lymph nodes at ACSHMC using GeneXpert, Ziehl–Neelsen (ZN) staining, and cytology.

## Materials and methods

### Study design and area

An institutional-based prospective cross-sectional study was conducted from May, 2022, to August, 2022. The study was conducted in the East Shoa zone, Oromia Regional State, Ethiopia, ACSHMC, Adama, Ethiopia. Adama city is located in the East-South direction about 100 km from the capital city of Ethiopia. Based on the 2021/2022 TB data, Adama Comprehensive Specialized Hospital Medical College (ACSHMC) has suspected a total of 1458 all forms of TB cases, from this 456 were confirmed TB Lymphadenitis in cytological diagnosis methods only. Gene X pert and AFB are not in use in the study site for the diagnosis of the disease.

### Study population

All patients with enlarged lymph nodes who visited ACSHMC, the pathology department for FNAC examination during the study period were considered for the study. The required sample size was determined using a single population proportion formula by considering prevalence reported from Bahirdar, Ethiopia (22.1%)^12^ and 95% confidence level. After considering 10% for the non-response rate, the total sample size was 291. We have used convenient sampling technique to recruit study participants.

### Eligibility criteria

#### Inclusion criteria

All patients who had enlarged lymph nodes and visited the pathology department at ACSHMC were included.

#### Exclusion criteria

The patient who had enlarged lymph node but the FNA sample is not sufficient for the laboratory test and patents not voluntary for this study were excluded.

### Study variables

Dependent variable: TB Lymphadenitis diagnosed by GeneXpert.

Independent variables: Socio-demographic and clinical characteristics.

### Data collection

For the collection of demographic and clinical characteristics, we used an interviewer-based structured questionnaire prepared after reviewing similar studies^12–14^. About 1.5–3 ml Fine Needle Aspirate (FNA) specimens were collected from enlarged lymph nodes to be diagnosed using GeneXpert^16^, ZN staining^17^, and Cytology^18^.

### Data quality control

The questionnaire was pre-tested on a population representing 5% of the sample size to check its consistency before the actual data collection. Each day, after data collection, collected data were checked and evaluated for completeness, accuracy, and clarity. The sample processing control contains non-infectious spores in the form of a dry spore cake that is included in each cartridge to verify adequate processing of TB. To control bias that could arise from laboratory investigation, smear microscopy were read by three professionals before the final issuance of the result.

### Statistical analysis

Data were entered into the computer using Epi Data version 3.1 and exported to SPSS version 25 (Statistical Package for Social Science) software for analysis. Bivariable and multivariable binary logistic regression were used to determine the association between dependent and independent variables. Initially, data were analysed using bivariable logistic regression; variables with *p* < 0.25 were further analyzed by multivariable logistic retrogression. The statistical significance was considered at a *p* < 0.05.

### Ethical consideration

Ethical clearance was obtained from the nationally registered ethical institutional review board of Hawassa University medical college (Ref No. IRB\148\14). All participants were informed of the purpose, risk, and benefit of the study and their participation was voluntary. Written informed consent was obtained from all participants. For minors, consent from the parents or legal guardians and assent from minors were obtained. The identifier of study participants were removed from all formats. All methods were carried out in accordance with relevant guidelines and regulation as mentioned by Declaration of Helsinki.

## Results

### Socio demographic characteristics

A total of 291 study participants with enlarged lymph nodes were included in this study, of which 121 (41.8%) were males. The mean age of study participants was 28 (SD ± 14.8). Most of the patients did not have history of treatment for TB (Table 1).

**Table 1:** Socio-demographic characteristics of participants with enlarged lymph nodes in patients Attending ACSHMC, Ethiopia, 2022 (N = 291).

Variables	Category	Frequency (%)
Sex	Male	121 (60)
	Female	170 (78)
Age group in years	< 14	54 (23)
	15–24	53 (28)
	25–34	101 (46)
	35–44	37 (18)
	45–54	27 (15)
	55–64	14 (5)
	> 65	5 (3)
Place of residence	Urban	177 (60.8)
	Rural	114 (39.2)
Marital status	Single	137 (47.1)
	Married	140 (48.1)
	Divorced	7 (2.4)
	Widowed	7 (2.4)
Occupation	Government employee	37 (12.7)
	Private job	54 (18.6)
	Farmer	28 (9.6)
	House wife	65 (22.3)
	Unemployed	15 (5.25)
	Student	89 (30.6)
	Daily laborer	3 (1)
Education	Unable to read and write	55 (18.9)
	Able to read and write	30 (18)
	Completed primary school	108 (37.1)
	Completed secondary school	35 (12.0)
	Completed College/university	63 (21.6)
Have monthly income	Yes	98 (33.7)
	No	193 (66.3)
Family size	1–3	136 (46.7)
	4–6	102 (35.1)
	> 7	53 (18.2)
Consumption of raw milk	Yes	118 (40.5)
	No	173 (59.5)

ACSHMC, Adama Comprehensive Specialized Hospital Medical College.

### Clinical characteristics

The common clinical presentations were fever (55%), weight loss (50.9%), night sweating (54.6%), and fatigue (52.6%). The most involved body sites (lymph nodes) were cervical (54.6%), axillary (11.3%) supraclavicular (10.3%), and submandibular (15.5%). The least affected lymph node were the auricular and inguinal lymph node with (1.4%), and (6.9%) respectively (Table 2).

**Table 2:** Clinical characteristics of patients with enlarged lymph nodes Attending ACSHMC, Ethiopia, 2022 (N = 291).

Clinical characteristics	Category	Frequency (%)
Chronic cough	Yes	99 (34.0)
	No	192 (66.0)
Fever	Yes	160 (55)
	No	131 (45)
Night sweating	Yes	159 (54.6)
	No	132 (45.4)
Loss of appetite	Yes	113 (38.8)
	No	178 (61.2)
Loss of weight	Yes	148 (50.9)
	No	143 (49.1)
Chest pain	Yes	94 (32.3)
	No	197 (67.7)
Shortness of breath	Yes	95 (32.6)
	No	196 (67.4)
Fatigue	Yes	153 (52.6)
	No	138 (47.4)
History of contact with TB patients	Yes	67 (23.0)
	No	171 (58.8)
	I don’t know	53 (18.2)
Confirmed pulmonary TB	Yes	53 (18.2)
	No	238 (81.8)
Treatment for TB	Yes	53 (18.2)
	No	238 (81.8)
BCG vaccination	Yes	82 (28.2)
	No	6 (2.1)
	I don’t know	203 (69.8)
HIV status	Positive	30 (10.3)
	Negative	182 (62.5)
	I don’t know	79 (27.1)
Aspirate type	Pus	220 (75.6)
	Soft	23 (7.9)
	Abscess	48 (16.5)
Lymph node involved	Cervical	159 (54.6)
	Axillary	33 (11.3)
	Submandibular	45 (15.5)
	Supraclavicular	30 (10.3)
	Inguinal	20 (6.9)
	auricular	4 (1.4)
Lymph size in centimeter (cm)	1–4	269 (92.4)
	5–10	22 (7.6)

BCG, Bacillus Calmette-Guérin; HIV, Human Immunodeficiency virus; TB, Tuberculosis; ACSHMC, Adama Comprehensive Specialized Hospital Medical College.

### Prevalence of TB lymphadenitis

From 291 participants with enlarged lymph nodes, 138 (47.4%) (95% CI 41.70–53.10) were MTB positive by the GeneXpert method. Whereas 100 (34.4%) (95% CI 28.94–39.85) and 123 (42.3%) (95% CI 36.63–47.00) were positive by ZN staining and cytology respectively. 85 (29.2%) were positive in all three methods; 102 (35%) positive by AFB and GeneXpert; 116 (39.9%) were positive by GeneXpert and cytology. Non-acid fast bacilli Bacteria were detected among 33 (11.3%) participants using Gram staining technique, out of which 32/33 (97%) were Gram—positive cocci and 1/33 (3%) were Gram—negative rods. Moreover, cytological diagnosis revealed 89 (30.6%) reactive lymphadenitis, 27 (9.3%) lymphoma, 16 (5.5%) chronic abscess, 6 (2.1%) malignancy and 8 (2.7%) carcinoma, 8 (2.7%) pyogenic, 5 (1.7%) benign, 3 (1.0%) hematoma 3 (1.0%) granuloma and 3 (1.0%) abscess.

### Rifampicin drug resistance gene

Out of 291 participants, 9 (3.1%) were infected with rifampicin-resistant genes positive *M. tuberculosis* while 3 (1.0%) of them were infected with *M. tuberculosis* which was rifampicin-intermediate. Overall, 9 out of 138 *M. tuberculosis* (6.5%) were positive for the rifampin-resistant gene.

### Factors associated with TB lymphadenitis

Variables such as place of residence, marital status, educational level, consumption of raw milk, and chronic cough showed a *p* < 0.25 in bivariable analysis and were selected for multivariable analysis. In multivariable analysis, TB lymphadenitis was significantly associated with chronic cough (Table 3).

**Table 3:** Bivariable and Multivariable analysis of factors associated with TB lymphadenitis among patients attending ACSHMC, Ethiopia, 2022 (N = 291).

Variable	TBLN positive n (%)	TBLN negative n (%)	COR (95% CI)	*p*-value	AOR (95% CI)	*p*-value
Marital status						
Single	63 (46)	74 (54)	0.142 (0.017–1.210)	0.074	0.860 (0.5511.344)	0.509
Married	66 (47.1)	74 (52.9)	0.149 (0.017–1.267	0.081		
Divorced	3 (42.9)	4 (57.1)	0.126 (0.009–1.671	0.116		
Widowed	6 (85.7)	1 (14.3)	1			
Place of residence						
Urban	73 (41.2)	104 (58.8)	0.529 (0.329–0.852)	0.009	0.698 (0.367–1.326)	0.272
Rural	65 (57)	49 (43)	1			
Educational level						
Unable to read & write	30 (54.5)	25 (45.5)	1.407 (0.681–2.908)	0.357	0.946 (0.757–1.181)	0.621
Able to read &write	18 (60)	12 (50)	1.759 (0.728–4.251)	0.210		
Primary	48 (44.4)	60 (55.6)	0.938 (0.502–1.614)	0.841		
Secondary	13 (37.1)	22 (62.9)	0.693 (0.297–1.614)	0.529		
College	29 (46)	34 (54)	1			
Raw milk consumption						
Yes	61(51.7)	57 (48.3)	1.334 (0.834–2.133)	0.228	0.868 (0.488–1.545)	0.631
No	77(44.5)	96 (55.5)	1			
BCG vaccination						
Yes	33 (40.2)	49 (59.8)	0.680 (0.404–1.144)	0.146	1.005 (0.731–1.381)	0.978
No	4 (66.7)	2 (33.3)	2.020 (0.362–1.274)	0.423		
I don’t know	101 (49.8)	102 (50.2)	1			
HIV status						
Positive	15 (50)	15 (50)	0.549 (0.235–1.286)	0.617	0.674 (0.429–1.060)	0.088
Negative	72 (39.6)	110 (60.4)	0.359 (0.208–0.622)	0.000		
I don’t know	51 (64.6)	28 (35.4)	1			
Chronic cough						
Yes	60 (60.6)	39 (39.4)	2.249 (1.370–3.690)	0.001	2.001 (1.142–3.508)	0.015
No	78 (40.6)	114 (59.4)	1			
Night sweating						
Yes	82 (51.6)	77 (48.4)	1.442 (0.908–2.300)	0.120	0.951 (0.539–1.679)	0.863
No	56 (42.4)	76 (57.6)	1			
Loss of appetite						
Yes	59 (52.2)	54 (47.8)	1.369 (0.853–2.197)	0.193	1.141 (0.660–1.971)	0.637
No	79 (44.4)	99 (55.6)	1			
Loss of weight						
Yes	76 (51.4)	72 (48.6)	1.379 (0.869–2.188)	0.173	0.925 (0.548–1.563)	0.771
No	62 (43.4)	81 (56.6)	1			
Shortness of breathing						
Yes	50 (52.6)	45 (47.4)	1.364 (0.834–2.229)	0.216	0.815 (0.441–1.507)	0.515
No	88 (44.9)	108 (55.1)	1			
Fatigue						
Yes	85 (55.6)	68 (44.4)	2.005 (1.255–3.202)	0.004	1.728 (0.977–3.058)	0.060
No	53 (38.4)	85 (61.9)	1			
Aspiration type						
Pus	104 (47.3)	116 (52.7)	0.558 (0.283–1.022)	0.058	0.826 (0.597–1.143)	0.249
Soft	4 (17.4)	19 (82.6)	0.126 (0.037–0.431)	0.001		
Abscess	30 (62.5)	18 (37.5)	1			

BCG, Bacillus Calmette-Guérin; HIV, Human Immunodeficiency virus; TBLN, TB lymphadenitis; COR, Crude Odds Ratio; AOR, Adjusted Odds Ratio; ACSHMC, Adama Comprehensive Specialized Hospital Medical College.

## Discussion

According to cytological studies, TB lymphadenitis is reported to be the common problem in Ethiopia^12,13,18^. However, these studies are mostly of retrospective type in nature and used cytology for the diagnosis of TB lymphadenitis^12,13,17^. The prevalence of TB lymphadenitis may vary depending on the method used for the diagnosis and the clinical background of the patients.

Many studies including our study revealed high prevalence of TB lymphadenitis among patients with enlarged lymph nodes as compared to other clinical conditions. In the current study, the overall prevalence of TB lymphadenitis using GeneXpert was 47.4%. Our finding is in line with the prevalence of TB lymphadenitis reported from Hawassa, Ethiopia (48.8%)^7^ and Addis Ababa, Ethiopia (49.3%)^15^. However, higher prevalence TB lymphadenitis was reported from Gondar, Ethiopia (65.7%)^13^, Jimma, Ethiopia (58.0%)^14^, Butajira, Ethiopia (72.8%)^19^ and Tanzania (69.5%)^20^. In contrast to our study, low prevalence of TB lymphadenitis was reported from Nigeria (24.45%)^21^ and Pakistan (44%)^22^. The variation might be due to socio-demographic factors and the laboratory method used. Some of the studies used cytological methods whereas others used molecular method which might be responsible for the differences observed.

The prevalence of TB lymphadenitis using ZN staining method was 34.4%. This finding is high compared to study conducted in Addis Ababa, Ethiopia (14.5%) which used a similar laboratory methods—ZN staining method^16^. A study conducted in a Sudan also reported low AFB positivity (9%) of lymphadenitis^23^.

According to the cytological examination (FNAC) of the present study, the prevalence of TB lymphadenitis was 42.3%. Reactive lymphadenitis was the second most frequent cause of enlarged lymph nodes. Lymphoma and the chronic abscess was the third and fourth frequent cause of enlarged lymph nodes respectively. The prevalence of TB lymphadenitis identified by cytological methods correlates with a study conducted in different parts of Ethiopia^7,12^. Another study from South Ethiopia showed a higher prevalence of TB lymphadenitis (68.7%) using cytological diagnosis^15^.

Overall, the performance of GeneXpert in diagnosing TB lymphadenitis was superior (47.4%) compared to the cytological method (42.3%) and ZN staining (34.4%). From the total study participants (N = 291), 29.2%, 35%, and 39.9% were positive for MTB by all three methods, AFB and GeneXpert, GeneXpert, and cytology respectively. Bacteria other than MTB were detected from 11.3% of participants with enlarged lymph nodes; the predominant bacteria were Gram-positive cocci. This indicates the role of other bacteria in lymphadenitis or it can be a super infection and need further study.

In contrast to a study conducted in Gondar^13^, in the current study male participants were more affected by lymphadenitis (48.8%) than their female counterparts (46.5%). This could be due to differences in lifestyle among males and females in different localities.

The majority of participants with enlarged lymph nodes were adults. 46% of participants within the age group of 25–34 years had enlarged lymph nodes whereas those within 15–24 years old accounted for 25%. This finding is inconsistent with other studies done in Ethiopia; where 25–34 years old are the commonest age group affected^14,15^. Review and Meta-analysis conducted in Africa indicated a high frequency of enlarged lymph nodes among individuals within the 20–40 years age group^24^.

Chronic cough was the only independent variable significantly associated with TB lymphadenitis. Patients who had a history of chronic cough had 2 times odds of developing TB lymphadenitis as compared to those who had no history of cough. This finding is in line with the finding reported from Jimma, Ethiopia^14^; however, it is not comparable to a study conducted in Gondar, Ethiopia^13^.

In this study, the cervical lymph node was found to be the most commonly affected site (54.6%). Similar finding was reported from northern parts of Ethiopia (57.1%)^12^. Hawassa, Ethiopia (57.1%)^7^, and Gondar, Ethiopia (47.5%)^13^. In addition, a report from India indicated a high prevalence of lymphadenitis involving cervical lymph nodes^4^.

The history of drinking raw milk among TB lymphadenitis cases was (21.0%). However, the history of drinking uncooked milk was not significantly associated with the occurrence of TB lymphadenitis. This finding is in line with the studies conducted in Addis Ababa, Ethiopia^15^.

In this study, 3.1% of participants were infected with rifampicin-resistant gene positive *M. tuberculosis* which is in line with the study conducted in Gondar, Ethiopia 3.9%^24^, India (2.9%)^25^, and Kenya (3.7%)^26^. A study conducted in Hawassa, Ethiopia among patients suspected of pulmonary TB revealed a prevalence of rifampicin resistance of 1.24% which is lower than the current study^27^. Out of 138 *M. tuberculosis* detected in this study, 9 were positive for the rifampin-resistant gene giving a proportion of 6.5%. Additionally, several studies reported a high prevalence of rifampicin resistance gene positive *M. tuberculosis* such as study from Nigeria (14.7%)^28^, India (5.4%)^4^, Addis Ababa, Ethiopia (9.9%)^29^, and Adigrat, Ethiopia (9.1%)^30^.

## Limitation of the study

As this study is a cross-sectional study, it will not establish the cause and effect. The participants might forget some variables which asks about their previous experience and might have led to recall bias. Use of convenient sampling technique might have introduced selection bias.

## Conclusions

Close to 50% of patients with enlarged lymph node were positive for TB lymphadenitis by GeneXpert in the study area. GeneXpert detected high number of TB lymphadenitis as compared to cytology and ZN staining technique. Chronic cough was significantly associated with TB lymphadenitis. The prevalence of rifampicin-resistant gene positive *M. tuberculosis* was relatively prevalent among patients with enlarged lymph node in the study area.

## Data Availability

All relevant data are available within the paper.
